# Comprehensive Analysis of Long Noncoding RNA Modified by m^6^A Methylation in Oxidative and Glycolytic Skeletal Muscles

**DOI:** 10.3390/ijms23094600

**Published:** 2022-04-21

**Authors:** Shanshan Wang, Baohua Tan, Liyao Xiao, Xinming Zhao, Jiekang Zeng, Linjun Hong, Jie Yang, Gengyuan Cai, Enqin Zheng, Zhenfang Wu, Ting Gu

**Affiliations:** 1 National Engineering Research Center for Breeding Swine Industry, College of Animal Science, South China Agricultural University, Guangzhou 510642, China; ws15907163745@163.com (S.W.); tanbaohua@stu.scau.edu.cn (B.T.); xlyao@stu.scau.edu.cn (L.X.); zxming@stu.scau.edu.cn (X.Z.); zengjiejang@stu.scau.edu.cn (J.Z.); linjun.hong@scau.edu.cn (L.H.); jieyang@scau.edu.cn (J.Y.); cgy0415@scau.edu.cn (G.C.); eqzheng@scau.edu.cn (E.Z.); 2 Guangdong Provincial Key Laboratory of Agro-Animal Genomics and Molecular Breeding, College of Animal Science, South China Agricultural University, Guangzhou 510642, China; 3 State Key Laboratory for Conservation and Utilization of Subtropical Agro-bioresources, Guangzhou 510642, China

**Keywords:** lncRNA, m^6^A methylation, muscle-fiber-type conversion, pig

## Abstract

N^6^-methyladenosine (m^6^A) is the most common modification in eukaryotic RNAs. Accumulating evidence shows m^6^A methylation plays vital roles in various biological processes, including muscle and fat differentiation. However, there is a lack of research on lncRNAs’ m^6^A modification in regulating pig muscle-fiber-type conversion. In this study, we identified novel and differentially expressed lncRNAs in oxidative and glycolytic skeletal muscles through RNA-seq, and further reported the m^6^A-methylation patterns of lncRNAs via MeRIP-seq. We found that most lncRNAs have one m^6^A peak, and the m^6^A peaks were preferentially enriched in the last exon of the lncRNAs. Interestingly, we found that lncRNAs’ m^6^A levels were positively correlated with their expression homeostasis and levels. Furthermore, we performed conjoint analysis of MeRIP-seq and RNA-seq data and obtained 305 differentially expressed and differentially m^6^A-modified lncRNAs (dme-lncRNAs). Through QTL enrichment analysis of dme-lncRNAs and PPI analysis for their cis-genes, we finally identified seven key m^6^A-modified lncRNAs that may play a potential role in muscle-fiber-type conversion. Notably, inhibition of one of the key lncRNAs, *MSTRG.14200.1*, delayed satellite cell differentiation and stimulated fast-to-slow muscle-fiber conversion. Our study comprehensively analyzed m^6^A modifications on lncRNAs in oxidative and glycolytic skeletal muscles and provided new targets for the study of pig muscle-fiber-type conversion.

## 1. Introduction

Skeletal muscle is a heterogeneous tissue, composed of different types of muscle fibers, with distinct contractile and metabolic characteristics [1]. Myosin heavy chain (MyHC) is the major contractile protein of skeletal muscle cells [2]. According to the electrophoretic analysis results of myosin heavy chain isoforms in adult mammals, muscle fibers are mainly divided into type I (MyHC I) and type II (MyHC IIa, MyHC IIb, and MyHC IIx/d) muscle fibers, and they are distinguished by their function, biochemical characteristics, and morphological characteristics [1,3,4,5]. Type I muscle fibers are rich in myoglobin and mitochondrial and mainly use lipids as an energy source and carry out aerobic respiration, while type IIb muscle fibers have a high content of glycogen and glucose, and mainly depend on glycolysis to obtain energy [6]. The other two types of muscle fibers, IIa and IIx/d, have intermediate biophysical properties [7]. In livestock, the composition of muscle fiber types is closely related to meat quality [8,9]. For example, Kim et al. reported that an increase in the proportion of IIb fiber decreases muscle pH and increases muscle cooking loss and lightness [10]. Different types of muscle fibers can transform into each other, and mutual conversion between MyHC subtypes follows an obligatory order: I ↔ IIx ↔ IIa ↔ IIb [11]. Muscle-fiber-type conversion is regulated by multiple signaling factors, such as myogenic regulation factors (MRFs) [12,13,14,15], nuclear factor of activated T cells (NFAT) [16], insulin-like growth factors (IGFs) [17,18], peroxisome proliferative activated receptor-γ activation of auxiliary factor 1 Alpha (PGC-1α) [19], and other unknown regulatory factors.

Long noncoding RNAs (LncRNAs), a class of RNAs with a length greater than 200 nucleotides that are unable to code proteins, have been found to participate in a variety of biological processes, including myogenic differentiation [20,21]. LncRNAs have also been reported to play an important regulatory role in muscle fiber conversion in livestock. For example, lncRNA *MyHC-IIa/X-as* promotes *MyHC-IIX* by sponging miR-130b, maintaining the fast fiber phenotype [22]. *LncRNA-FKBP1C* regulates muscle-fiber-type switching by affecting the stability of *MYH1B* [23]. LncRNAs function through diverse mechanisms. In the nucleus, lncRNAs act on regulating the transcription program via chromatin interaction and remodeling and establishing spatial organization of nuclear compartments through scaffolding. In the cytoplasm, lncRNAs mediate signal transduction pathways, posttranscriptional control of gene expression, and translational processes [24].

N^6^-methyladenosine (m^6^A) is the most abundant internal modification on mRNAs [25]. It is a reversible RNA modification that prefers to occur on a consensus motif RRACH (in which R represents A or G, and H represents A, C, or U) near mRNA stop codon and in long internal exons [25]. m^6^A is introduced by a nuclear methyltransferase complex, including METTL3, METTL14, WTAP, and RBM15 (or RBM15B), which have been designated as m^6^A “writers” [26], and can be removed by m^6^A demethylases, such as FTO and ALKBH5, which have been designated as m^6^A “erasers” [26]. Furthermore, multiple proteins, including YTHDF1, YTHDF2, YTHDF3, and YTHDC1, acting as m^6^A “readers”, can bind to modified sites and regulate a variety of post-transcriptional processes [27]. The functions of m^6^A in mRNA include nuclear transport, splicing, stability, and translation [28]. Many lncRNAs have also been found to be modified by m^6^A, and m^6^A modification affects these lncRNAs’ expression levels and mechanisms of action. For example, *MALAT1* can regulate the variable splicing and gene transcription of pre-mRNA. It is found that *MALAT1* contains multiple m^6^A modification sites. m^6^A can change the binding ability of *MALAT1* to protein by changing its RNA structure, and as such, affect the splicing and transcription function of *MALAT1* [29]. *Xist* is also highly methylated by m^6^A, knocking out m^6^A methyltransferase METTL3 inhibits *Xist*-mediated X chromosome silencing [30]. However, the effect of m^6^A modification on lncRNAs is far from clear. Recently, using methylated-RNA immunoprecipitation sequencing (MeRIP-seq), Xie et al. revealed the temporal expression profile and m^6^A methylation status of lncRNAs during skeletal myogenesis [31]. However, little is known about the m^6^A methylation status of lncRNAs and the regulation of m^6^A on lncRNAs during muscle-fiber-type conversion.

In this study, we identified 5607 novel lncRNAs in oxidative and glycolytic skeletal muscles using RNA-seq, and further disclosed a large number of m^6^A sites and specific m^6^A modification patterns in lncRNAs using MeRIP-seq. We highlight the potential role of m^6^A-modified lncRNAs in muscle fiber conversion by regulating the expression of the muscle-development-related gene in cis. Our results reveal the tissue-specific expression and m^6^A methylation status of lncRNAs in different types of muscle fibers, which provides a basic reference for further study of the regulation of m^6^A on lncRNAs.

## 2. Results

### 2.1. Difference in Phenotypic Traits and m^6^A-Related Gene Expression Levels between Oxidative and Glycolytic Skeletal Muscles

**Figure 1:**
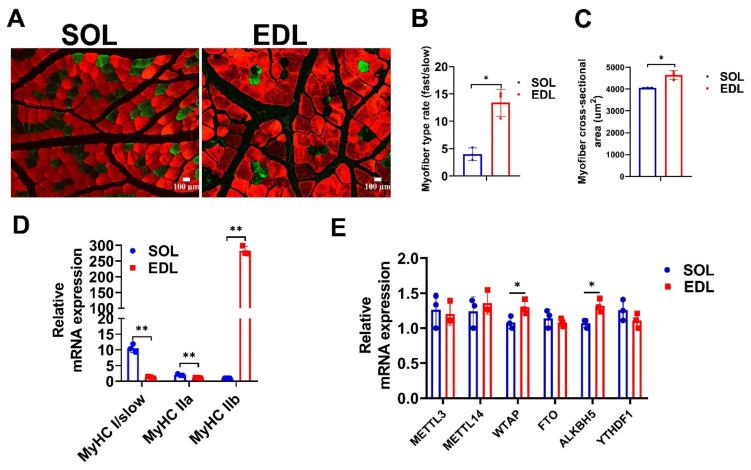
Detection of expression levels of muscle-fiber-type related genes and m^6^A-related genes. (**A**) Representative images of MyHC I (green) and MyHC IIb (red) immunofluorescent staining in SOL and EDL muscle sections. Scale bar: 100 μm. (**B**) Quantification of the cross-sectional area of SOL and EDL muscle sections in (**A**). (**C**) Statistical analysis of fast and slow muscle fibers of SOL and EDL muscles in (**A**). (**D**) qPCR experiment detects the expression level of *MyHC I*, *MyHC IIa*, and *MyHC IIb* in SOL and EDL. (**E**) qPCR experiment detects the expression level of m^6^A-related genes *METTL3*, *METTL14*, *WATAP*, *FTO*, *ALKBH5*, and *YTHDF1* in SOL and EDL. The relative RNA expressions were standardized to that of the control gene β-actin. Data represent mean ± SD of three independent biological replicates. * *p* < 0.05, ** *p* < 0.01.

First, we detected the phenotypic traits of soleus (SOL) and extensor digitorum longus (EDL) muscles of a 6-month-old Duroc pig. The immunofluorescence staining results of the glycolytic muscle-fiber marker MyHC IIb and the oxidative muscle-fiber marker MyHC I showed that the composition of glycolytic muscle fiber in EDL was almost three times that of SOL (Figure 1A,B), and the average cross-sectional area of individual myofiber in EDL was larger than that of SOL (Figure 1C). Moreover, the mRNA expression of *MyHC IIb* was higher in EDL than in SOL, while *MyHC I* and *MyHC IIa* mRNA expressions were higher in SOL than in EDL (Figure 1D). The above results indicate that EDL is a typical glycolytic skeletal muscle, while SOL is a typical oxidative skeletal muscle. We also detected the expression of m^6^A writers (*METTL3*, *METTL14*, and *WTAP*), erasers (*FTO* and *ALKBH5*), and read (*YTHDF1*) genes in SOL and EDL. The qPCR results showed that the mRNA levels of *WTAP* and *ALKBH5* were extraordinarily lower in SOL than in EDL (Figure 1E).

### 2.2. Dynamic Changes of lncRNAs’ Transcriptome in Oxidative and Glycolytic Skeletal Muscles

**Figure 2:**
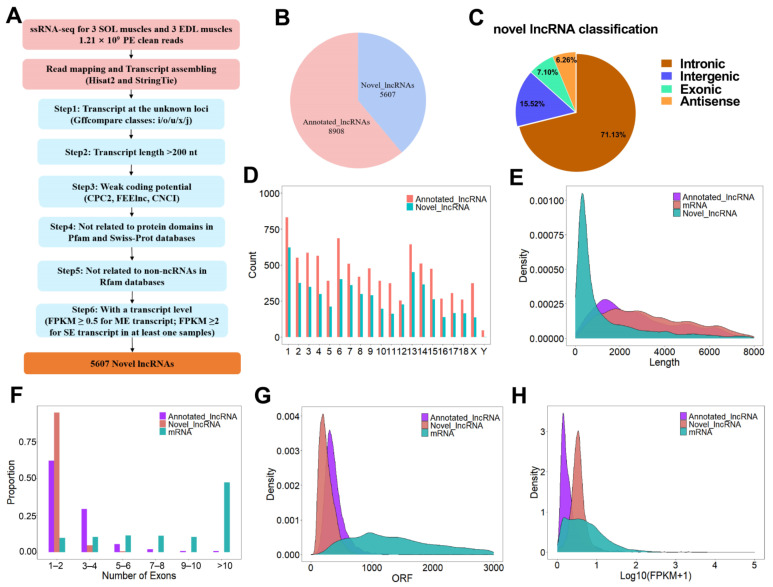
Identification and characterization of lncRNAs identified in SOL and EDL. (**A**) Bioinformatics pipeline for novel lncRNAs identification. nt: nucleotide; ME: multiple exons; SE: single exons. (**B**) Pie chart showing the proportion of novel lncRNAs identified in our study and previously annotated pig lncRNAs. (**C**) Classification of novel lncRNAs identified in this study. (**D**) Chromosome distribution of lncRNAs. (**E**–**G**) Distribution of transcript lengths (**E**), exon number (**F**), ORF length (**G**) in lncRNAs and mRNAs. (**H**) Distribution of expression level (showing log10 (FPKM + 1)) in lncRNAs and mRNAs.

LncRNAs have recently been reported to have important roles in regulating muscle development and muscle-fiber conversion. To understand the genetic basis of lncRNA-regulated skeletal-muscle-fiber development, we performed RNA sequencing (MeRIP-seq input library) on the SOL and EDL muscles of 6-month-old Duroc pigs. A total of 530,385,924 raw reads were generated from three SOL and three EDL muscle samples. After filtering out reads containing adapters and reads of low quality, a total of 445,953,950 properly paired mapped reads were mapped to the pig reference genome (Sscrofa 11.1.94), accounting for 84.08% of the raw reads. After a series of filtering steps, shown in Figure 2A, a total of 5607 novel lncRNAs were obtained in SOL and EDL (Figure 2B and Table S1). It is worth noting that 71.13% (3988), 7.1% (398), 15.52% (870), or 6.26% (351) of the novel lncRNAs were intronic lncRNAs, exonic lncRNAs, lincRNAs (long intergenic non-codingRNAs), or antisense lncRNAs, respectively (Figure 2C). An analysis of genomic location distribution indicated that the lncRNAs were distributed on all chromosomes, without obvious chromosome preference (Figure 2D). To understand the characterization of the identified novel lncRNAs, we made comparisons of the gene structure and expression patterns among novel lncRNAs, annotated lncRNAs, and mRNAs. The results showed that the novel lncRNAs and annotated lncRNAs shared common characteristics, including shorter transcript length, fewer exons, shorter ORF, and lower expression compared with mRNAs (Figure 2E–H). 

**Figure 3:**
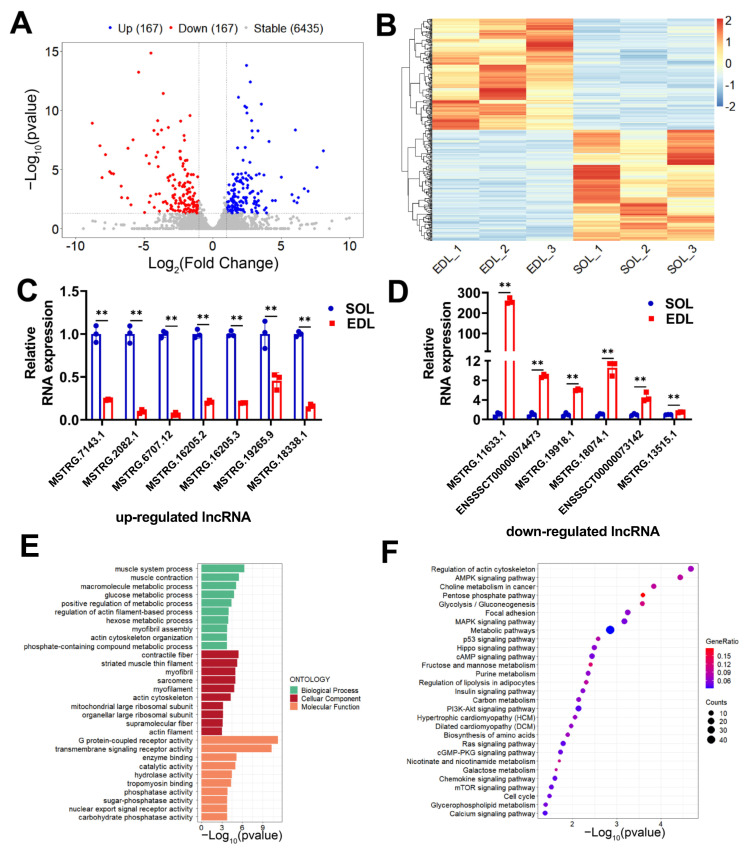
Screening and functional enrichment analysis of DE lncRNAs in SOL compared with EDL. (**A**) Volcano plot showing the expression profile of DE lncRNAs between SOL and EDL. Blue dots \\SOL; grey dots represent lncRNAs with stable expression in both tissues. (**B**) Hierarchical clustering heatmap showing the expression profile of DE lncRNAs between SOL and EDL. (**C**,**D**) 7 upregulated (**C**) and 6 downregulated (**D**) DE lncRNAs were randomly selected and verified via qPCR. (**E**) GO enrichment analysis of the nearest target genes of DE lncRNAs. (**F**) KEGG pathway analysis of the nearest target genes of DE lncRNAs. The relative RNA expressions were standardized to that of the control gene β-actin. Data represent mean ± SD of three independent biological replicates. ** *p* < 0.01.

A total of 6769 (including 5607 novel lncRNAs and 1162 annotated lncRNAs) expressed lncRNAs were used for further differential expression analysis (Table S1). We identified 334 differentially expressed lncRNAs (DE lncRNAs) between SOL and EDL (Table S2), among which, 167 were upregulated and 167 were downregulated in SOL (Figure 3A). Hierarchical clustering analysis showed that the expression patterns of DE lncRNAs were, as expected, considerably different between the SOL group and EDL group, while there were small differences among the three biological replicates in each group (Figure 3B). To verify the reliability of our RNA-seq results, 13 DE lncRNAs, including 7 upregulated and 6 downregulated, were randomly selected to validate their expression differences between SOL and EDL by qPCR. The results showed that all detected lncRNAs displayed either higher or lower transcript abundance in SOL or EDL, which was consistent with that of the RNA-seq results (Figure 3C,D). Given that lncRNAs have no encoding potential, their functions are achieved through the regulation of target genes. Hence, we determined the cis target genes to explore the potential function of DE lncRNAs. GO enrichment and KEGG pathway analysis on the cis target genes of DE lncRNAs were conducted. GO analysis results suggested the important role of DE lncRNAs in regulating muscle-fiber switching, as numerous processes related with skeletal-muscle-fiber properties were enriched. For example, muscle system processes, muscle contraction, glucose metabolic process, and myofibril assembly were significantly enriched in the BP term; contractile fiber, myofibril, and mitochondrial large ribosomal subunits were remarkably enriched in the CC term, and G protein-coupled receptor activity, transmembrane signaling receptor activity, enzyme binding, catalytic activity, and phosphatase activity were observably enriched in the MF term (Figure 3E and Table S3). KEGG pathway enrichment analysis showed that pathways related to muscle-fiber properties were enriched, including glycolysis/gluconeogenesis, mTOR-signaling pathways, metabolic pathways, insulin-signaling pathways, AMPK-signaling pathways, and PI3K-Akt-signaling pathways (Figure 3F and Table S3). Taken together, our study identified numerous novel and differentially expressed lncRNAs in different muscle-fiber types and highlighted the potential regulation of lncRNAs in muscle-fiber conversion.

### 2.3. Overall Features of lncRNAs m^6^A Methylation in Oxidative and Glycolytic Skeletal Muscles

**Figure 4:**
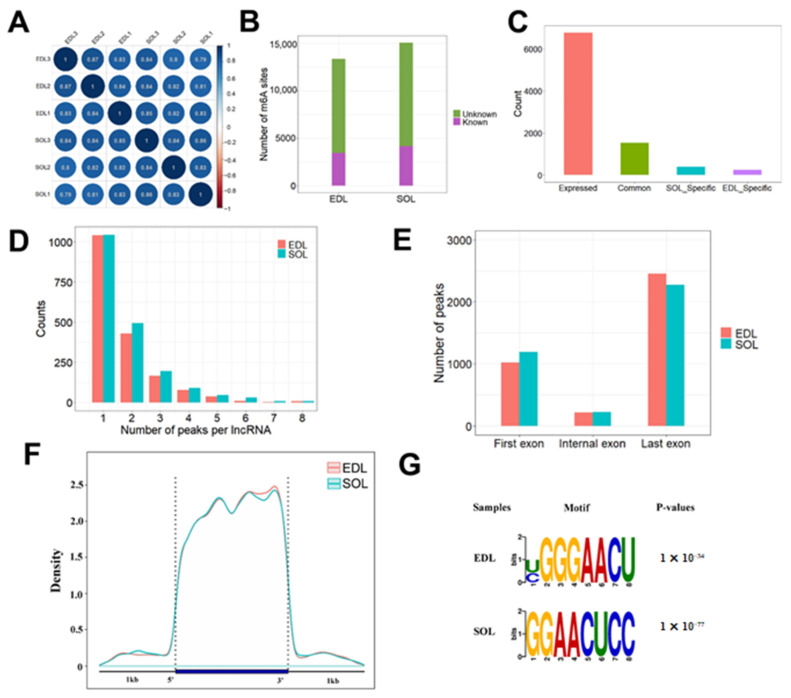
Overall features of lncRNAs m^6^A methylation in oxidative and glycolytic skeletal muscles. (**A**) Pearson correlation analysis of MeRIP-seq data between each pair of samples. The samples were hierarchically clustered. The intensity of the color represented the similarity, and the black outlining was drawn according to the hierarchical cluster. (**B**) The number of m^6^As found in each tissue. Any m^6^A site that did not overlap with any site in RMBase were labelled as ‘unknown’. (**C**) Histogram showing the distribution of lncRNAs with m^6^As. Expressed: expressed lncRNAs; Common: lncRNAs with m^6^As in two tissues; SOL_-_specific: lncRNAs with m^6^As in SOL only; EDL_-_specific: lncRNAs with m^6^As in EDL only. (**D**) The distribution of peak number of m^6^A-modified lncRNAs. (**E**) Metagene profile of enrichment of m^6^As in lncRNAs. (**F**) Histogram showing the distribution of m^6^As in the three regions of lncRNAs. (**G**) The enriched consensus motif of m^6^As in lncRNAs.

To reveal the m^6^A methylation profile of lncRNAs expressed in oxidative and glycolytic skeletal muscles, we performed methylated-RNA immunoprecipitation and sequencing (MeRIP-seq) and obtained an average of 80,387,850 properly paired mapped reads per sample, with a greater than 89% mapping rate. Pearson’s correlation analysis showed that the three biological replications of the same samples were clustered well (Figure 4A), suggesting that our sequencing data within the same group were highly consistent. In total, we identified 15,071 and 13,380 m^6^A peaks (hereafter m^6^As) in SOL and EDL, respectively. Among these identified peaks, 10,925 m^6^As in SOL and 9949 m^6^As in EDL were novel m^6^As that have not been reported in RMbase (Figure 4B). We further focused on the m^6^As located in lncRNAs. We identified 2888 m^6^As in 1925 expressed lncRNAs in SOL and 2527 m^6^As in 1782 expressed lncRNAs in EDL; among them, 1534 lncRNAs were m^6^A-modified in two tissue types, and 391 and 248 lncRNAs were only m^6^A-modified in SOL and EDL, respectively (Figure 4C and Table S4). Among the 391 lncRNAs specifically modified by m^6^A in SOL, 11 lncRNAs were specifically expressed in SOL and 9 lncRNAs were specifically expressed in EDL. Among the 248 lncRNAs specifically modified by m^6^A in EDL, 13 lncRNAs were specifically expressed in SOL and 23 lncRNAs were specifically expressed in EDL (Table S4). We checked the number of m^6^As in each lncRNA and found that most lncRNAs contained 1–2 m^6^As in SOL and EDL tissues (Figure 4D). We then analyzed the peak distribution for lncRNAs, and found that m^6^As were preferentially enriched in the last exon of the lncRNAs (Figure 4E). Furthermore, the distribution pattern of m^6^A on the lncRNA transcripts with a 1 kb flanking region was roughly uniform, but with a slight increase in the 3′ end (Figure 4F), as reported in a previous study [32]. 

To identify the common sequence elements of the m^6^A peaks on lncRNAs, we used HOMER software to identity a consensus motif with default parameters that will generate motifs ranging from three to eight bases. We found the enriched motif containing the well validated consensus m^6^A motif RRACH [33,34] in the SOL and EDL groups (Figure 4G), which were similar to the previously identified m^6^A motif in the lncRNAs [31]. All the above analyses could provide a fundamental reference for the m^6^A epitranscriptome in skeletal muscle with different types of muscle fiber. 

### 2.4. Association Analysis of m^6^A with lncRNAs Expression

**Figure 5:**
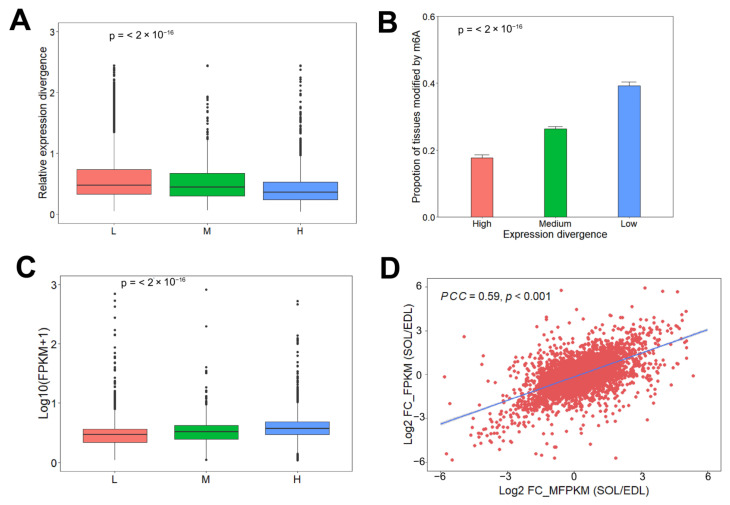
Association analysis of m^6^A with lncRNAs expression. (**A**) Relative expression divergence of lncRNAs in the L set (4596 lncRNAs), M set (639 lncRNAs) and H set (1534 lncRNAs). Significance was evaluated by a two-sided Mann–Kendall trend test, *p* <  2.2 × 10^−16^. (**B**) The lncRNAs were clustered into Low (1693 lncRNAs), Median (3383 lncRNAs), and High (1693 lncRNAs) groups according to the quantile of the expression divergence. Significance was evaluated by a two-sided Mann–Kendall trend test, *p* <  2.2 × 10^−16^. (**C**) Expression level in lncRNAs in the L set (4596 lncRNAs), M set (639 lncRNAs) and H set (1534 lncRNAs). Significance was evaluated by a two-sided Mann–Kendall trend test, *p* <  2.2 × 10^−16^. (**D**) Scatter plot showing the positive correlation between m^6^A levels and expression level of lncRNAs between EDL and SOL. FC: Fold change; PCC: Pearson correlation coefficient.

To further examine the role of m^6^A methylation in lncRNAs’ expression dynamic, we classified the 6769 expressed lncRNAs in SOL and EDL into three sets, according to the proportion of transcripts with m^6^A peaks in two tissues, L: lncRNAs without m^6^A peaks in two tissue types, M: lncRNAs with m^6^A peaks in only one tissue type, and H: lncRNAs with m^6^A peaks in both tissue types. We found that lncRNAs in the H sets had the most stable expression levels, while lncRNAs in the L sets showed the least stable expression levels (Figure 5A). We next clustered the 6769 lncRNAs into Low, Median, and High groups, according to the quantile of the expression divergence. We found that lncRNAs with more stable expression levels were also more likely to have a higher proportion of transcripts modified by m^6^A methylation in two tissue types (Figure 5B). These results indicated that m^6^A methylation was positively correlated with lncRNAs’ expression homeostasis. Furthermore, we explored the association between m^6^A methylation abundance and lncRNAs’ expression levels. We found that the expression level of m^6^A-modified lncRNAs was higher than that of non-m^6^A-modified lncRNAs, and lncRNAs with m^6^A methylation in both tissue types had the highest expression levels (Figure 5C). We then examined the correlation of lncRNAs’ expression levels with m^6^A methylation levels; Pearson correlation coefficient analysis results showed that lncRNAs’ m^6^A levels were positively correlated with their expression levels (R = 0.72, *p* < 0.001) (Figure 5D). These results demonstrate that m^6^A may play a role in lncRNAs transcription activation.

### 2.5. Conjoint Analyses of MeRIP-seq and RNA-seq Data

**Figure 6:**
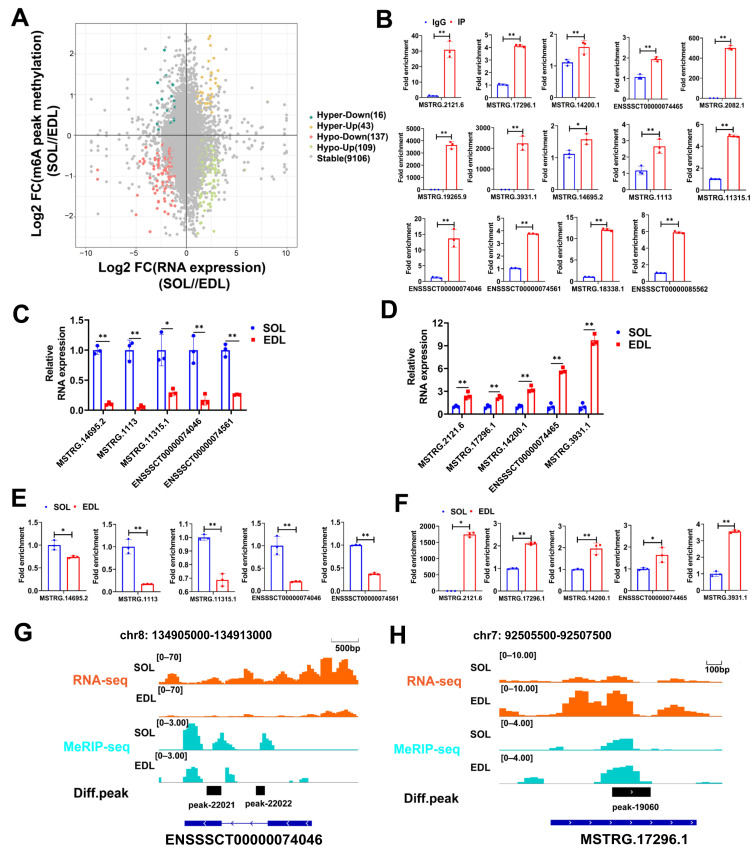
Conjoint Analyses of MeRIP-Seq and RNA-Seq Data. (**A**) Four-quadrant graph showing the distribution of lncRNAs with a marked change in both RNA expressions and m^6^A methylation levels in SOL compared with EDL. Different colors were used to identify representative lncRNAs. (**B**) MeRIP-qPCR validated 14 m^6^A-methylated lncRNAs. IgG was used as negative control. (**C**,**D**) MeRIP-qPCR results showed that 5 Hyper-Up lncRNAs of the 14 m^6^A-methylated lncRNAs have higher m^6^As enrichment in SOL than EDL (**C**), 5 Hypo-Down lncRNAs of the 14 m^6^A-methylated lncRNAs have higher m^6^As enrichment in EDL than SOL (**D**); data were normalized by IgG. (**E**) qPCR results showed that the expression of 5 Hyper-Up lncRNAs were increased in SOL compared with EDL. (**F**) qPCR results showed that the expression of 5 Hypo-Down lncRNAs were decreased in SOL compared with EDL. (**G**,**H**) Genome browser tracks showing RNA-seq (orange) and MeRIP-seq (light blue) data at lncRNA *ENSSSCT00000074046* (**G**, scale bar: 500 bp) and *MSTRG. 17296.1* (**H**, scale bar: 100 bp) loci in SOL and EDL. The relative RNA expressions were standardized to that of the control gene β-actin. Data represent mean ± SD of three independent biological replicates. * *p* < 0.05; ** *p* < 0.01.

To explore the potential role of m^6^A on lncRNAs, we performed conjoint analysis of MeRIP-seq and RNA-seq data. Of the 334 DE lncRNAs in Figure 3A, 305 (88.9%) lncRNAs were also shown to be differentially m^6^A modified, and these lncRNAs were referred to as dme-lncRNAs. A high proportion of DE lncRNAs modified by m^6^A indicated that m^6^A might participate in muscle-fiber-type conversion by regulating the transcription of lncRNAs. Among the 305 dme-lncRNAs, 152 upregulated lncRNAs were significantly hyper-methylated (43; Hyper-Up) or hypo-methylated (109; Hypo-Up) in SOL, and153 downregulated lncRNAs were significantly hyper-methylated (16; Hyper-Down) or hypo-methylated (137; Hypo-Down) in SOL (Figure 6A). To confirm the significantly differentially m^6^A-modified lncRNAs, we randomly selected 14 significantly differentially m^6^A-modified lncRNAs and performed methylated RNA immunoprecipitation, followed by real-time PCR (MeRIP-qPCR). The results showed that all selected lncRNAs were remarkably enriched in the m^6^A group, compared to the IgG control (Figure 6B). Moreover, we compared the m^6^A enrichment and expression differences of the above 14 lncRNAs in SOL and EDL. We finally validated 10 (5 Hyper-Up lncRNAs and 5 Hypo-Down lncRNAs) out of 14 lncRNAs, with the same change in the m^6^A and expression levels between SOL and EDL (Figure 6C–F), which was consistent with the analysis results, indicating that the analysis results were highly reliable. As shown in the IGV, lncRNA *ENSSSCT00000074046* had higher m^6^As enrichment in SOL, and its expression level was also higher in SOL (Figure 6G), while lncRNA *MSTRG. 17296.1* had a higher m^6^As enrichment in EDL, and its expression level was also higher in EDL (Figure 6H). Taken together, these findings suggest that the m^6^A enrichment change in lncRNAs may affect their expression levels.

### 2.6. Identification of Key Muscle-Fiber-Types-Related lncRNAs

Previous studies have shown that different muscle fibers are closely related to animals’ economic traits, such as meat quality and muscle growth [35,36]. To identify m^6^A-modified lncRNAs that regulate muscle-fiber-type conversion, we conducted an enrichment analysis by mapping dme-lncRNAs to the QTL regions in pigs. The animal QTLdb (PigQTLdb) database has a total of 31,455 pig quantitative trait loci (QTLs), representing 695 different traits [37]. We found 162 dme-lncRNAs were located in 2919 QTLs; among them, “meat and carcass traits”-related QTLs accounted for the largest proportion (68.8%). In addition, QTLs related to “healthy traits”, “production traits”, “reproduction traits”, and “exterior traits” accounted for 14.1%, 10.5%, 3.8%, and 2.8%, respectively. We mainly focused on QTLs associated with meat-associated traits, and we identified 49 dme-lncRNAs that were associated with “meat and carcass traits”; among them, some were closely associated with muscle fiber characteristics, such as meat color, fatty acid content, pH, flavor, and enzyme activity.

**Figure 7:**
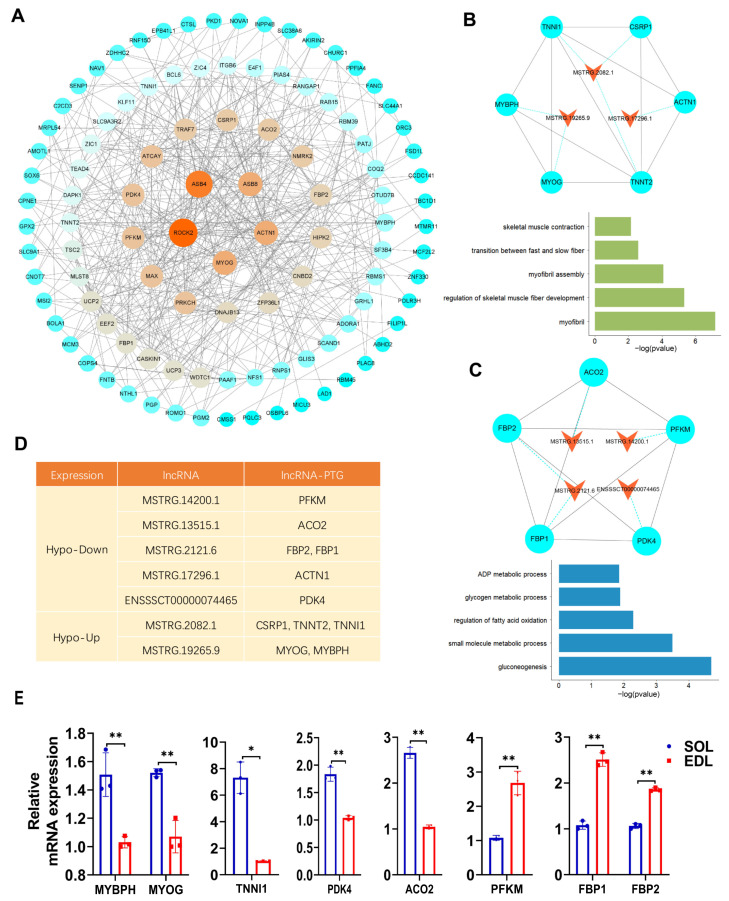
QTL analysis and functional enrichment of dme-lncRNAs. (**A**) Protein–protein interaction (PPI) network of PTGs of lncRNAs visualized by using Cytoscape. The size and color of circles represented the degree of interaction between the genes. (**B**,**C**) Above: the top two clusters from PPI network identified by using MCODE. Bottom: GO enrichment analysis of PTGs in corresponding cluster. The orange triangle node represented lncRNAs, the blue cycle node represented PTGs. Each pair of PTGs and lncRNAs was indicated by blue dotted line. The interaction of PTGs was indicated by black solid line. (**D**) The information of lncRNAs and their adjacent mRNAs from (**E**). (**E**) qPCR result showing the adjacent mRNA expressions in SOL and EDL. The relative mRNA expression was standardized to that of the control gene β-actin. Data represent mean ± SD of three independent biological replicates. * *p*< 0.05; ** *p* < 0.01.

It has been reported that lncRNAs can regulate their neighboring gene transcription in cis [38]. To further explore the potential function of the 49 dme-lncRNAs, we identified their potential cis target genes (PTGs) by searching for expressed protein-coding genes around 100 kb of these lncRNAs. In this way, we found 119 cis PTGs for 49 dme-lncRNAs. Then, we performed protein–protein interaction (PPI) analysis for 119 cis PTGs, and found the muscle-related genes, such as *MYOG*, *ACTN1*, that were the key codes in the PPI network (Figure 7A), suggesting these dme-lncRNAs may regulate muscle-related genes to participate in the process of muscle-fiber-type conversion. Furthermore, we screened out the nodes and conducted MCODE analysis to identify highly connected clusters in a large PPI network (Table S5). We selected the genes in the top two clusters for GO enrichment analysis and found that they were significantly enriched in the pathways related to muscle-fiber properties and energy metabolism (Figure 7B,C). We investigated six PTGs involved in muscle-fiber property pathways, corresponding to three dme-lncRNAs, *MSTRG.2082.1*, *MSTRG.19265.9*, and *MSTRG.17296.1*, and five PTGs involved in energy metabolism pathways, corresponding to four dme-lncRNAs, *MSTRG.13515.1*, *MSTRG.14200.1*, *MSTRG.2121.6*, and E*NSSSCT00000074465* (Figure 7D and Table S6). The cis PTGs of seven dme-lncRNAs might play an important role in the conversion of muscle-fiber types. In particular, *MSTRG.2082.1* was involved in the regulation of *TNNI1*, which is a marker gene of slow muscle fiber. *MSTRG.13515.1* was involved in the regulation of *ACO2*, which is a part of the citric acid cycle and metabolizes α-ketoglutarate, a product of glutamine oxidation [39]. Furthermore, we detected the mRNA expression of PTGs of these seven dme-lncRNAs in SOL and EDL; the qPCR results showed that the mRNA expression of *MYBPH*, *MYOG*, *TNNI1*, *PDK4*, and *ACO2* was upregulated in SOL, while *PFKM*, *FBP1*, and *FBP2* mRNA expression was downregulated in SOL (Figure 7E). Notably, the mRNA expression of *MYBPH*, *MYOG*, *TNNI1*, *PFKM*, *FBP1* and *FBP2* was consistent with their nearby lncRNAs, *MSTRG.19265.9* and *MSTRG.2082.1*, and *MSTRG.14200.1* and *MSTRG.2121.6*, respectively. In contrast, the mRNA expression of *ACO2* and *PDK4* was opposite to their nearby lncRNAs, *MSTRG.13515.1* and *ENSSSCT00000074465*, respectively (Figure 7E). In summary, we identified seven key m^6^A-modified lncRNAs that may affect muscle-fiber-type conversion by positively or negatively regulating their cis target genes.

### 2.7. Inhibition of MSTRG.14200.1 Reduced PSCs Differentiation and Stimulated Fast-to-Slow Muscle-Fiber Conversion

**Figure 8:**
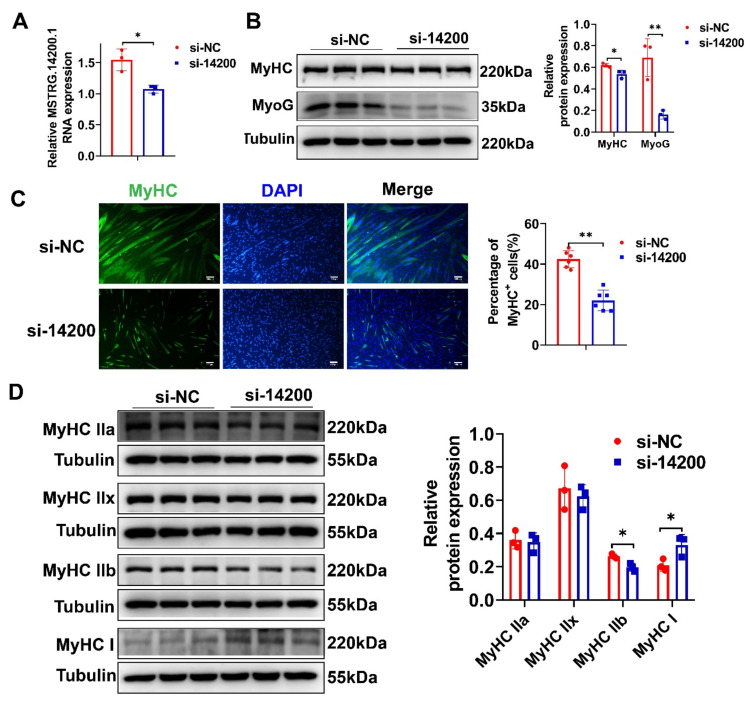
Inhibition of *MSTRG.14200.1* reduced PSC differentiation and stimulated fast-to-slow muscle-fiber conversion. (**A**) qPCR result showed that *MSTRG.14200.1* expression was significantly reduced. (**B**) Western blotting result showed that the protein expression levels of MyoG and MyHC were remarkably decreased after *MSTRG.14200.1* knockdown. (**C**) Immunofluorescence staining result of MyHC showed that knockdown of *MSTRG.14200.1* significantly reduced the proportion of MyHC^+^ cells. Scale bar: 200 μm. (**D**) Western blotting result showed that *MSTRG.14200.1* knockdown notably inhibited MyHC IIb protein expression and enhanced MyHC I protein expression. The relative RNA and protein expression were standardized to that of the control gene β-actin and Tubulin, respectively. Data represent mean ± SD of three independent biological replicates. * *p* < 0.05; ** *p* < 0.01.

To further explore the role of lncRNAs in pig skeletal muscle satellite cell (PSCs) differentiation and muscle-fiber-type conversion, we chose lncRNA *MSTRG.14200.1*, which has a higher expression level in EDL than in SOL, for further loss-of-function assays. The expression level of *MSTRG.14200.1* was successfully knocked down using siRNA (Figure 8A). By Western blotting and immunofluorescence staining, we found that *MSTRG.14200.1* knockdown significantly decreased MyoG and MyHC protein expression (Figure 8B) and MyHC^+^ cells proportion (Figure 8C), indicating the *MSTRG.14200.1* knockdown inhibited PSC differentiation. The *MSTRG.14200.1* knockdown also remarkably reduced MyHC IIb protein expression, while increasing MyHC I protein expression (Figure 8D), which showed that the *MSTRG.14200.1* knockdown stimulated fast-to-slow muscle-fiber conversion. All the above results demonstrate that *MSTRG.14200.1* has an important role in promoting PSC differentiation and inducing slow-to-fast muscle-fiber conversion.

## 3. Discussion

Skeletal muscle fiber formation and the conversion of different types of muscle fibers are complex processes, regulated by many factors. The current research on skeletal muscle-fiber-type conversion is mainly focused on signal transduction pathways, protein-coding gene regulation, and nutritional intervention [40,41,42]. Recent reports have also shown the important roles of lncRNAs in skeletal muscle development and muscle-fiber-type conversion [21,22,23]. A more recent report has revealed the m^6^A methylation status of lncRNAs during skeletal myogenesis [31]. However, it is unclear whether lncRNAs’ m^6^A methylation is involved in muscle-fiber-type conversion. In the present study, we hypothesized that lncRNAs might also be regulated by m^6^A and participate in skeletal muscle-fiber-type conversion. Hence, we comprehensively identified differentially m^6^A-methylated lncRNAs in oxidative and glycolytic skeletal muscle by MeRIP-seq. To the best of our knowledge, this study is the first systematic evaluation of m^6^A methylomes profiles of lncRNAs in oxidative and glycolytic skeletal muscle. Our work provides a valuable resource for the future study of the regulation and function of m^6^A modification on lncRNAs in muscle-fiber-type conversion.

The size of the pig genome is roughly the same as that of the human and mouse genome. However, fewer lncRNAs were annotated in pigs compared with the above two species [43,44,45]; this indicates that a great quantity of pig lncRNAs have not been discovered. In this study, we identified 5607 novel lncRNAs, which broaden the annotation of the pig lncRNAs. We found that the transcript length, number of exons, ORF length, and gene expression abundance of the novel identified lncRNAs are similar to previously annotated lncRNAs, but differ from the mRNAs, in accordance with those of other studies [46,47]. We used three kinds of coding potential software (CNCI, FEElnc, and CPC2) to exclude transcripts with protein-coding potential [48,49,50]. We noted that some lncRNAs also have long putative ORF, equivalent to mRNA ORF, such as those with ORF ≥ 500 nt; these lncRNAs may protein code mRNAs containing unknown protein domains. Furthermore, we identified 334 DE lncRNAs between SOL and EDL through RNA-seq, providing key candidate lncRNAs involved in the regulation of skeletal muscle-fiber-type conversion.

Studies have demonstrated that m^6^A is a ubiquitous modification in mRNAs and plays a key role in regulating gene expression [34]. m^6^A modification in lncRNAs has also attracted the attention of researchers. In this study, we identified a large number of m^6^A peaks in lncRNAs, in both SOL and EDL muscles, through MeRIP-seq, indicating lncRNAs are extensively modified by m^6^A. We found typical m^6^A consensus motifs within lncRNAs, as previously identified in lincRNAs [51]. m^6^A was evenly enriched in the lncRNA body, increased slightly at the 3′ end, and more located in the last exon of lncRNA, which was similar to a previous report [32,52]. These results show that the m^6^A peaks identified in our study are credible. m^6^A methylation in mRNA affects nuclear transport, splicing, stability and translation of mRNA [28]. m^6^A methylation in lncRNAs can also regulate their expression. For example, METTL3-mediated m^6^A methylation modification, resulting in *LINC00958* upregulation by stabilizing its RNA transcript and the high level of *LINC00958,* led to the poor overall survival of hepatocellular carcinoma patients [53]. Another study reported that the *METTL14* knockdown can abolish the m^6^A level of *XIST* and enhanced *XIST* expression, and m^6^A-methylated *XIST* can also be recognized by YTHDF2 to mediate the degradation of *XIST* [54]. In the present study, we systematically analyzed the role of m^6^A methylation in lncRNA expression dynamics, and found the lncRNAs’ m^6^A levels were positively correlated with their expression levels, indicating m^6^A methylation may positively regulate these lncRNAs’ expressions. m^6^A-reader proteins are responsible for recognizing m^6^A sites and play a role in regulating RNA stability. The m^6^A-reader protein YTHDF2 was shown to mediate RNA decay [55,56] and other reader proteins, such as IGF2BP1/2/3, YTHDC1, and YTHDF1/3, and can strengthen RNA stability [30,56,57]. Thus, we speculate that the lncRNAs, whose expression levels are positively regulated by m^6^A in our study, may be mainly recognized and bound by IGF2BP1/2/3, YTHDC1, or YTHDF1/3 and, thus, enhance their expression. However, further research is needed to confirm this hypothesis, and more efforts should be undertaken to clarify how m^6^A methylation affects lncRNAs expression.

Certain types of skeletal muscle fibers are known to exert a pivotal function in determining the meat quality of livestock after death, such as meat color, drip loss, and pH [58]. To identify lncRNAs that were specifically involved in muscle-fiber-type conversion, we took advantage of the animal QTLdb (PigQTLdb) database, and performed a correlation analysis by mapping dme-lncRNAs to the QTL regions related to pig-meat-related traits. This analysis allowed us to identify 49 dme-lncRNAs closely associated with muscle-fiber-type characteristics, such as meat color, fatty acid content, PH, flavor, and enzyme activity. A large number of studies have shown that the biological function of lncRNAs can be predicted by evaluating the relevant cis genes [46]. Therefore, we further predicted the function of the 49 dme-lncRNAs by searching for their PTGs, and we found 119 cis PTGs of 49 dme-lncRNAs. These genes were further used for lncRNAs’ functional annotation and enrichment analysis. Finally, we identified seven dme-lncRNAs, whose PTGs were enriched in muscle-fiber properties and energy metabolism (for example, *MSTRG.2082.1* paired with *CSRP1*, *TNNT2*, and *TNNI1)*. Of note, *TNNI1*, encoding the slow skeletal muscle isoform, is specifically expressed in slow muscle fibers and has been used as a model gene to study the specific expression mechanism of slow fibers [59,60]. Our qPCR results also found that both *MSTRG.2082.1* and *TNNI1* expressions were upregulated in SOL compared with in EDL, indicating *MSTRG.2082.1* co-located with *TNNI1* may positively affect the expression of *TNNI1* and, thus, regulate the conversion of skeletal muscle-fiber types. Besides, *MSTRG.14200.1* paired with *PFKM*, as *PFKM* is a key regulator of glycolysis, encodes a muscle subtype of phosphofructose kinase, called phosphofructose kinase 1 [61]. This is a strong candidate for skeletal muscle gene expression, associated with glycemic traits [62]. Interestingly, we found both *MSTRG.14200.1* and *PFKM* have a higher expression in EDL than in SOL, indicating *MSTRG.14200.1* may play a role in regulating glycolytic muscle fiber through enhancing *PFKM* expression. However, whether the seven dme-lncRNAs play a functional role in skeletal muscle-fiber-type conversion needs further systematic functional research.

In summary, we analyzed the differential expressions and m^6^A-methylation profiles of lncRNAs in oxidative and glycolytic skeletal muscles, and identified seven m^6^A-modified lncRNAs that may play an important role in muscle-fiber-type conversion. In addition, we preliminarily verified that lncRNA *MSTRG.14200.1* can promote PSC differentiation and slow-to-fast muscle-fiber conversion. Our study provides the resource of m^6^A-modified lncRNAs profile for future studies on the function and mechanism of m^6^A-modified lncRNAs, and it opens up a new way for the study of RNA epigenetics in muscle-fiber-type conversion.

## 4. Materials and Methods

### 4.1. Sample Preparation

Three 6-month-old Duroc pigs were obtained from the breeding pig farm of Guangdong Wen’s Foodstuffs Group Co., Ltd. (Yunfu, China). Samples of soleus muscles (SOL) and extensor digitorum longus muscles (EDL) were immediately put into liquid nitrogen and then stored at −80 °C for RNA extraction or fixed in 4% paraformaldehyde for histology staining. All animal experiments were conducted based on the National Research Council Guide for the Care and Use of Laboratory Animals and approved the Animal Care and Use Committee of the South China Agricultural University, Guangzhou, China.

### 4.2. m^6^A-seq and RNA-seq Library Preparation

Total RNA of three 6-month-old Duroc pigs was extracted using TRIzol reagent (Invitrogen, Carlsbad, CA, USA) in accordance with the manufacturer’s instructions. RNA amount and purity were quantified using NanoDrop ND-1000 (NanoDrop, Wilmington, DE, USA). The RNA integrity was assessed by Bioanalyzer 2100 (Agilent, Santa Clara, CA, USA) and confirmed by agarose gel electrophoresis. Ribosomal RNA was depleted from total RNA using the Epicentre Ribo-Zero Gold Kit (Illumina, San Diego, CA, USA), and the ribosomal-depleted RNA was fragmented using Magnesium RNA Fragmentation Module (Cat.e6150, NEB, Ipswich, MA, USA) at 86 °C for 7 min. Then the cleaved RNA fragments were incubated with m^6^A-specific antibody (No. 202003, Synaptic Systems, San Jose, CA, Germany) and Dynabeads antibody Coupling Kit (Thermo Fisher Scientific, Waltham, MA, USA) in IP buffer (50 mM Tris-HCl, 750 mM NaCl and 0.5% Igepal CA-630) for 2 h at 4 °C. The IP RNA then underwent a series of processing, including reverse transcription to produce cDNA, synthesis of second-strand DNA, incorporation of dUTP solution into the second strand, addition of A-base at the end of each strand, and finally PCR amplification to form a library with fragment size of 300 ± 50 bp. Both the Input samples without immunoprecipitation and the m^6^A IP samples libraries were sequenced on an illumina Novaseq™ 6000 (LC-Bio Technology CO., Ltd., Hangzhou, China) to produce paired-end 150 bp reads.

### 4.3. Identification of lncRNAs

The clean data were obtained by removing reads containing adapters, reads containing over 10% of poly (N) by fastq from the raw data [63]. Hisat2 was used to map the clean reads to reference genome (Ensembl Sscrofa 11.1.94) under chain-specific parameters: “rna-strandness RF” [64]. Unique mapped reads with a mapping quality greater than 20 using samtools [65]. The mapped paired-reads from each library were assembled with StringTie (v2.1.1) [66] to construct and identify transcripts through a reference-based approach, and calculate fragments per kilo-base of exon per million fragments (FPKM) of lncRNAs and coding genes. The assembled transcripts were compared with known gene models using gffcompare [67], and transcripts with class codes ‘i’, ‘j’, ‘o’, ‘u’, and ‘x’ were selected. We further removed transcripts that were shorter than 200 nt in length. The Coding-Non-Coding Index (CNCI) [48], Flexible Extraction of LncRNAs (FEElnc) [49] and Coding Potential Calculator 2 (CPC2) [50] were used to evaluate the coding potential of filtered transcripts with default parameters. The transcripts without coding potential on the above three software were retained. The remaining transcripts were compared with the Swiss-Prot (https://www.ebi.ac.uk/uniprot, (accessed on 16 July 2021), Pfam protein (http://pfam.xfam.org, (accessed on 16 July 2021) and Rfam (http://rfam.xfam.org, (accessed on 16 July 2021) databases to exclude the potential protein-coding genes and known ncRNAs including tRNA, ribosomal RNA (rRNA), small nuclear RNA (snRNA) and small nucleolar RNA (snoRNA). Finally, the transcripts with FPKM ≥ 0.5 (2 for single-exon transcripts) at least in one sample were considered as “novel lncRNAs” (Figure 2A). Both datasets of known and novel lncRNAs were combined into the final lncRNAs set used in this research.

### 4.4. Differential Expression Analysis

mRNAs and lncRNAs with ≥0.5 FPKM in at least one library were considered expressed and were used for further analysis. Differential gene expression analysis between two groups was performed using the R package DESeq2 [68]. LncRNAs with adjusted *p*-value < 0.05 using Benjamini–Hochberg and log2 fold change (log2FC) ≥ |1| were considered differentially expressed lncRNAs. 

### 4.5. Functional Enrichment Analysis

To predict the functions of the lncRNAs, the expressed mRNAs within 100 kb of lncRNAs were defined as cis target genes and submitted to functional enrichment analysis. Gene ontology (GO) analyses and Kyoto Encyclopedia of Genes and Genomes (KEGG) pathway analyses were performed using PANTHER [69] and KOBAS-i [70], respectively. The GO terms and pathways categories with *p* value < 0.05 were considered significant. The pig quantitative trait loci (QTLs) database was downloaded from the Animal QTLdb (PigQTLdb) (http://www.animalgenome.org/QTLdb/pig.html, (accessed on 19 October 2021). The command “intersect” in bedtools was used to acquire lncRNAs enriched in QTL traits locus [71].

### 4.6. Establishment of PPI Network

PPI network was established by the STRING (v10.5) [72], and those experimentally validated interactions with a combined score > 0.4 were selected. The screened networks were visualized by the Cytoscape 3.6.1 [73]. Then, the “Molecular Complex Detection” (MCODE), a clustering algorithm identifying locally densely connected regions in a large PPI network based on node-weighting arithmetic, was performed to establish PPI network modules with parameters (Degree cutoff = 2, Node score cutoff = 0.3, k-core = 2, Max. Depth = 100).

### 4.7. MeRIP-Seq Analysis

The mapped reads from the IP and Input libraries were fed into the R package exomePeak2 (v1.6.0) for calling peaks under default parameters settings, and identifying differential peaks (adjusted *p*-value < 0.05) [74]. Identified peaks were annotated by intersecting with gene architecture using bedtools and custom shell script. Peaks located at expressed lncRNAs were selected for downstream analysis. Peaks that did not overlap with any m^6^A modification site in RMBase [75] were determined to be unknown. The distribution of m^6^As in lncRNAs was characterized by Guitar R package (v1.16.0) [76]. The DREME tool in the MEME suite (http://meme-suite.org/tools/dreme, (accessed on 10 August 2021) was used to discover relatively short (up to 8 bp) motifs that were enriched within a set of target sequences (m^6^A peak sequences) [77]. The FPKM of Input and IP samples were calculated by StringTie. The m^6^A enrichment levels of lncRNAs were represented as MFPKM (MFPKM = FPKM_IP/FPKM_INPUT) averaged in the three biological replicates. The Pearson correlation analysis and principal component analysis (PCA) of sequencing data were performed using DeepTools [78]. Read coverage of regions was visualized via the Integrative Genomics Viewer (IGV) [79].

### 4.8. MeRIP Assay

The MeRIP experiment procedure was performed as mentioned in ‘m^6^A-seq library preparation’, except that total RNA was not subjected to ribosomal RNA (rRNA) removal and fragmentation. The Input RNA and immunoprecipitated RNA from SOL and EDL muscle tissues were reverse-transcribed to produce cDNA by SuperScript™ II Reverse Transcriptase (Invitrogen, Carlsbad, CA, USA), and analyzed using real-time quantitative PCR (qPCR). 

### 4.9. Real-Time Quantitative PCR (qPCR)

Total RNA of PSCs and tissues was obtained using Trizol reagent (Invitrogen, Carlsbad, CA, USA) and reverse-transcribed to create cDNA by Evo M-MLV RT Kit (AG, Changsha, China). qPCR was performed on an ABI QuantStudio 7 Flex system (Thermo Fisher Scientific, Waltham, MA, USA) using PowerUp^TM^ SYBR^TM^ Green Master Mix (Thermo Fisher Scientific, Waltham, MA, USA). The Ct (2^−ΔΔCt^) method was used to analyze relative RNA expression. All primers used for qPCR in this study are listed in Table S7.

### 4.10. Histology Staining

The fixed tissues were embedded in paraffin and cut into 4 μm thin slices. Then the sections were used for immunofluorescence staining of MyHC I (BF-D5; DSHB; Iowa City, IA, USA) and MyHC IIb (BF-F3; DSHB; Iowa City, IA, USA). Immunofluorescence staining on paraffin muscle sections was performed in accordance with previous reports [80]. The images were captured using a Nikon ECLIPSE Ci microscope (Nikon, Tokyo, Japan) and analyzed using the ImageJ software (NIH, Bethesda, MD, USA).

### 4.11. Porcine Skeletal Muscle Satellite Cells Isolation and Culture

Porcine skeletal muscle satellite cells (PSCs) were isolated from leg muscles of gilts within a week. The gilt was sacrificed, washed with flowing water, and disinfected with 75% alcohol, then the leg muscles were removed and placed in PBS containing 1% penicillin-streptomycin (Gibco, Carlsbad, CA, USA). The muscles were cut into pieces and digested in 2 mg/mL collagenase I (Sigma-Aldrich, St. Louis, MO, USA) at 37 °C for 2 or 3 h with shaking, until the mixture became uniform. Digestion was then stopped and the mixture was in turn filtered through 100-, 200-, and 400-mesh sieves. The cells were differential-adhesion cultured in RPMI 1640 medium supplemented with 20% FBS (Gibco, Carlsbad, CA, USA), 1% non-essential amino acids (Gibco, Carlsbad, CA, USA), 1% GlutaMAX (Gibco, Carlsbad, CA, USA), 0.5% chicken embryo extract (Gemini Bio, Sacramento, CA, USA), 4 ng/mL basic fibroblast growth factor (Thermo Fisher Scientific, Waltham, MA, USA) and 1% penicillin-streptomycin for 2 h to remove fibroblasts. The supernatant containing purified cells was then transferred to a cell culture bottle coated with collagen for proliferating culture at 37 °C and 5% CO_2_. 

For the differentiation of PSCs, when the cells reached 70–80% confluence, the proliferating medium was replaced with RPMI 1640 containing 2% horse serum (Gibco, Carlsbad, CA, USA). 

### 4.12. siRNA Synthesis, and Cell Transfection

siRNA for *MSTRG.14200.1* and negative control (NC) were designed and synthesized by GenePharma (Shanghai, China). siRNA oligos sequences were as follows: *MSTRG.14200.1* (sense 5′- GCCUACUUAGUGCAGAAACTT’), NC (sense 5′- UUCUCCGAACGUGUCACGUTT-3′). siRNA oligos transfection of PSCs were performed with Lipofectamine 3000 reagent (Invitrogen, Carlsbad, CA, USA) according to the manufacturer’s protocol. 

### 4.13. Western Blotting

Total protein of PSCs was extracted with RIPA buffer containing 1% PMSF (Beyotime, Shanghai, China), and the protein was then incubated at 95 °C for 5 min for denaturation in 6× protein loading buffer. Western blotting was performed according to previous reports [81]. The primary antibodies used include: MyHC I (sc-53089; Santa Cruz; Delaware, Santa Cruz, CA, USA), MyHC IIb (A15293; ABclonal; Wuhan, China), MyHC IIa (A15292; ABclonal; Wuhan, China), MyHC IIx (A6935; ABclonal; Wuhan, China), MyHC (sc-376157; Santa Cruz; Delaware, Santa Cruz, CA, USA), MyoG (sc-12732; Santa Cruz; Delaware, Santa Cruz, CA, USA), and β-Tubulin (GB11017B; Servicebio; Wuhan, China). The secondary antibodies used include: Goat Anti-Mouse IgG (A0216; Beyotime; Shanghai, China) and Goat Anti-Rabbit IgG (A0208; Beyotime; Shanghai, China). Protein band intensity was quantified using ImageJ.

### 4.14. Cell Immunofluorescence Staining

PSCs were fixed in 4% paraformaldehyde and permeabilized with 0.5% Triton X-100 for 30 min, respectively. After three washes, the cells were blocked in QuickBlock™ Blocking Buffer (Beyotime, Shanghai, China) for 2 h, incubated with a primary antibody overnight and incubated with a secondary antibody for 1 h, in turn. Finally, the nuclei were stained with DAPI reagent. Images of at least three random fields of view were captured using a Nikon ECLIPSE Ti microscope (Nikon, Tokyo, Japan) and quantified using ImageJ. The primary antibody was MyHC (sc-376157; Santa Cruz; Delaware, Santa Cruz, CA, USA). The secondary antibody was FITC-labeled Goat Anti-Mouse IgG (A0568; Beyotime, Shanghai, China).

### 4.15. Statistical Analysis

Statistical analyses for the results of qPCR were performed by two-tailed, unpaired Student’s *t*-test using SPSS software (IBM, Armonk, NY, USA) and the histogram plots were generated by GraphPad Prism 8.0.2 software (GraphPad Software, San Diego, CA, USA). Data were presented as means ± standard deviation (SD) in at least triplicate. A *p* < 0.05 was considered as statistically significant. The significance was marked as * *p* < 0.05 and ** *p* < 0.01.

## Data Availability

The datasets presented in this study can be found in online repositories. The name of the repository and accession number can be found below: https://www.ncbi.nlm.nih.gov/bioproject/PRJNA810786/, (accessed on 3 March 2022), PRJNA810786.

## Supplementary Materials

The following supporting information can be downloaded at: https://www.mdpi.com/article/10.3390/ijms23094600/s1.
